# Impact of the COVID‐19 Pandemic on Oral Health Behaviors Among Children in Iran

**DOI:** 10.1002/cre2.70010

**Published:** 2024-11-11

**Authors:** Mahtab Memarpour, Faranak Razmjouei, Fatemesadat Fayazi, Azade Rafiee, Mehrdad Vossoughi

**Affiliations:** ^1^ Oral and Dental Disease Research Center School of Dentistry Shiraz University of Medical Sciences Shiraz Iran

**Keywords:** attitude, awareness, COVID‐19, dental caries, oral hygiene

## Abstract

**Objectives:**

Parents are responsible for their children's oral health. This study evaluated parents' knowledge, attitudes, and performance on their children's oral health before and after an educational intervention and the impact of COVID‐19 on these variables.

**Material and Methods:**

This quasi‐experimental study included 227 children aged 6–8 years who were referred to 11 Shiraz medical centers from July to December 2021. Data were collected from questionnaires and interviews. After completing the questionnaire, oral health education was provided immediately and then monthly thereafter using dental models and pamphlets. After 3 months, a questionnaire assessed the education's effectiveness. Data were analyzed using independent *t*‐tests, one‐way analysis of variance (ANOVA), paired *t*‐tests, and Pearson's correlation.

**Results:**

A total of 163 participants (71.80%) completed all phases, including the second questionnaire. Participants showed moderate knowledge (77.30%), attitude (89.00%), and performance (80.40%). Neither knowledge nor attitude before the intervention had a significant association with demographic characteristics. Parents' education, number of children, and maternal employment were significantly associated with performance (*p* = 0.002, *p* = 0.040, *p* < 0.001, respectively). The intervention significantly enhanced these variables (*p* < 0.001). In terms of COVID‐19, 72.40% of participants expressed good knowledge about transmission, 52.80% showed moderate concern, and 54% had poor performance. Parental knowledge, attitude, and performance changed significantly following the intervention (*p* = 0.030, *p* < 0.001, *p* < 0.001, respectively).

**Conclusions:**

Educational intervention significantly improved parents' knowledge, attitudes, and performance toward children's oral health. Because of the poor performance of parents during the COVID‐19 pandemic, we recommend the implementation of continuing education and preventive oral health programs.

## 1 Introduction

Maintenance of oral health and preventive dentistry are among the most important goals of pediatric dentistry. Although cariogenic microorganisms, fragmented carbohydrates, and teeth (host) are the main risk factors for dental caries, they can be eliminated by preventive techniques. Oral health care such as brushing, dental flossing, and the use of chemical agents are effective homecare methods for caries prevention. Other preventive strategies include professional fluoride therapy and pit and fissure sealants, which are provided by health care providers (Anil and Anand 2017; Kazeminia et al. 2020; Pakkhesal et al. 2021).

Health behaviors are influenced by awareness, attitude, and performance. Parents are the behavioral models for their children. As role models, they encourage their children to establish healthy habits to prevent diseases. Studies that assessed parents' knowledge, attitudes, and performance in terms of children's oral health reported a relationship between parents' education, preventive behavior, and oral health status of school children (Basir, Khanehmasjedi, and Khanehmasjedi 2022; Dumitrescu et al. 2022). In contrast, one study reported that oral health educational and behavioral intervention programs did not improve these variables for refugee families in America (Alrashdi et al. 2021).

In 2020, the World Health Organization (WHO) announced that the acute respiratory syndrome coronavirus (COVID‐19) was a global pandemic (Cucinotta and Vanelli 2020). Although children were as susceptible to this infection as adults, the clinical manifestations were different (Vidya et al. 2021). During the COVID‐19 pandemic, extensive closures and restrictions led to the provision of medical services for emergency cases. One study revealed that the COVID‐19 pandemic reduced dental treatments, and this resulted in a lack of access to public dental services, delays in elective treatments, and a failure to use preventive dentistry; this led to a heavy demand on the health care system after the pandemic (Chisini et al. 2021). Besides, the quarantine during the pandemic resulted in changes in sleep patterns and unhealthy eating habits that included frequent use of snacks (Cheikh Ismail et al. 2021). One study revealed a tremendous increase in self‐medication practices for children's dental problems during the COVID‐19 pandemic (Sen Tunc et al. 2021). Another study assessed mothers' knowledge of COVID‐19 to evaluate their attitudes and fears about dental visits during the pandemic. Mothers perceived the dental clinics as a risky place for contracting the virus and were unlikely to take their children to the dentist except for emergency cases (Farsi and Farsi 2021). Furthermore, another study reported the lack of knowledge among parents about close contact with asymptomatic patients, dentistry practices in transmission, and the role of children in transmission (Ceyhan, Kirzioglu, and Yildirim 2022).

To our knowledge, there have been limited comprehensive studies that assess the levels of knowledge, attitudes, and performance of parents in relation to children's oral and dental health during the COVID‐19 pandemic. Therefore, the aim of the present study was to assess the levels of these variables before and after an oral health educational intervention. The impact of the COVID‐19 pandemic on the aforementioned indices was also evaluated. In this study, we used a combination of face‐to‐face interviews, a patient education dental model, and oral health care pamphlets. Also, participants were interviewed via phone or video call to discuss their children's oral health. The effectiveness of this method was assessed at the end of the follow‐up period.

## 2 Study Population and Methodology

### 2.1 Ethics

The Research Ethics Committees of the School of Dentistry, Shiraz University of Medical Sciences approved this study. The methodology was conducted in adherence to pertinent rules and legislation. Informed consent was obtained from all participants in the study according to the Declaration of Helsinki.

### 2.2 Study Design

This was a quasi‐experimental study. Early enrollment included 227 children, aged 6–8 years, who were referred to pediatricians in 11 public and private medical centers in four areas of Shiraz. The study was conducted from July to December 2021. The data were collected by a dentist who used questionnaires, face‐to‐face interviews, a patient education dental model, and oral health care pamphlets. The interviewer (F.F.) was trained by initially interviewing 50 parents under the supervision of two professors. A thorough literature review was used as the basis for the questions. The questionnaire started with a brief introduction that explained the aim of the research and the ethical issues that pertained to the answers.

### 2.3 Sample Size Determination

The primary sample size was calculated to be 160 participants, which was determined according to the expected percentage of participants who knew the goal of brushing (*p* = 80%), precision (*d* = 10%), 95% confidence level, and by taking into consideration a design effect of 2.5 for the cluster sampling method. However, due to the unknown size of the selected clusters, we included a total of 227 people in the study.

### 2.4 Inclusion and Exclusion Criteria

The study included 6‐ to 8‐year–old children whose parents provided informed consent for data sharing. The parents also participated at the beginning of the study and follow‐up. The parents were assured that their survey responses were confidential. The research excluded children with special diseases, serious medical conditions, physical or mental disabilities, and those who declined to participate. In addition, incomplete questionnaires were excluded from the final assessment.

### 2.5 Questionnaire

The questionnaire consisted of 72 questions divided into seven sections: demographics; parents' knowledge, attitudes, and performance about children's oral health; and these variables during the COVID‐19 pandemic (File S1).

1. The demographics of the parents and participating children were determined by 10 questions about the participant's age, gender, date of birth, number of children in the family, birth order of the child, parents' educational levels, and parents' occupation.
2. Oral health knowledge was assessed using 16 questions. Of these, 11 were scored according to a 5‐point Likert scale that ranged from strongly agree (ranked as 5) to strongly disagree (ranked as 1). There were also four multiple‐choice questions and one short‐answer question. Incorrect responses received a score of 0, whereas correct responses were scored as 1. The questions evaluated the parents' knowledge of predisposing factors to dental caries, preventative measures and the definition of dental plaque and its related problems. All question scores were added to the participants' knowledge scores. This section measured awareness from 0 to 18.
3. Attitudes toward oral health were by 10 questions that were scored on the same 5‐point Likert scale ranging from strongly agree (ranked as 5) to strongly disagree (ranked as 1). Strongly agree and disagree received a score of 1 and the others received a score of 0. The questions focused on the parents' perspectives on the necessity of dental treatments and the importance of preserving primary teeth. The total attitude score was measured between 0 and 10.
4. We used seven questions to determine the oral health practice of the children. This section was scored by a 4‐point Likert scale that ranged from always (Score 3), often (Score 2), sometimes (Score 1), and never (Score 0). Each question was scored based on the categories mentioned earlier (from 0 to 3). Also, nine multiple‐choice questions were designed and calculated as mentioned before. Correct responses received 1 point per response. In questions that had multiple correct answers, points were provided based on the number of correct items selected. In addition, six descriptive questions were developed and the percentages of the respondents were compared. The questions focused on performance caries preventive behaviors that included the frequency of brushing and dental visits. The performance score ranged from 0 to 39.

In addition, the impact of the COVID‐19 pandemic on the aforementioned three variables was assessed. On a 5‐point Likert scale, five questions assessed parents' awareness of the probability of children being infected with the COVID‐19 virus in dental offices. Three 5‐point Likert scales evaluated the parents' attitudes with regard to their concerns about COVID‐19. Four questions with three possible answers (increased, decreased, or unchanged) rated the performance of parents in maintaining their children's oral health during the COVID‐19 pandemic. Also, two descriptive questions assessed their performance, and the percentages of respondents were compared. The performance rating was assigned a value between 0 and 4.

Overall, the scores from knowledge, attitude, and practice that were lower than one‐third of the attainable score were considered to be poor. The scores from one‐third to two‐thirds of the attainable score were considered to be moderate, whereas the scores of two‐thirds of the attainable score or more were defined as good. Higher ratings indicated higher oral and dental health awareness, attitudes, or practices in the participants.

Once participants' parents completed the initial questionnaire, oral health instruction was provided to the children and their parents by demonstrating tooth brushing and dental flossing on a dental model. In addition, four educational pamphlets were discussed and distributed to the parents. After the initial interview, the training phases were repeated each month for 3 months by phone or video call. At the end of the study, the parents completed a follow‐up questionnaire to evaluate the effectiveness of the oral health instruction. A phone number was provided to answer parents' questions or to arrange necessary dental treatments for children during the pandemic and the research period.

### 2.6 Reliability and Validity of the Questionnaires

Cronbach's alpha was computed for knowledge, attitude, and practice questionnaires for the first 50 participants. The alpha values were 0.75, 0.71, and 0.78 for knowledge, attitude, and practice, respectively, and indicated an acceptable level of internal consistency. The alpha values were 0.73, 0.70, and 0.72 for the variables with respect to COVID‐19. Reliability was assessed using the test–retest technique on selected participants. Fisher's exact and Wilcoxon's signed‐rank tests were used to examine the similarity of responses to dichotomous and Likert‐scale questions, respectively.

An expert panel that included eight pediatric dentists assessed the content validity of the questionnaires. The content validity ratio (CVR) was calculated for each item. Only items with a CVR value of 0.76 or higher were used in the questionnaire.

### 2.7 Statistical Analysis

Data were described using frequency, percentage, mean, and standard deviation (SD). Data were analyzed with SPSS software for Windows, Version 22.0 (IBM Corp., Armonk, NY, USA). The independent samples t‐test, one‐way analysis of variance (ANOVA), paired sample *t*‐test, and Pearson's correlation coefficient were used for statistical analyses. The *p* values less than 0.05 indicated statistical significance. The ranges of each score were calculated.

## 3 Results

Out of 227 parent participants, 163 (71.80%) completed the study. The mean age of children was 7.00 ± 0.79 years and their mothers had a mean age of 29.56 ± 5.63 years. Table 1 shows the participants' demographic characteristics.

**Table 1:** Study participants' demographics.

Variable	*N* (%)
Children's age (years)	
6	52 (31.9)
7	64 (39.2)
8	52 (31.9)
Sex	
Male	89 (54.6)
Female	74 (45.4)
Number of children	
1	52 (31.9)
2	79 (48.5)
≥ 3	32 (19.6)
Children's birth order	
1	90 (55.2)
2	56 (34.4)
≥ 3	17 (10.40)
Fathers' education level	
Undocumented	0
Less than diploma	23 (13.60)
Diploma	53 (32.60)
Associate's degree	16 (9.90)
Bachelor's degree	34 (21)
Master's degree	31 (19.1)
PhD degree	6 (3.7)
Mothers' education level	
Undocumented	0
Less than diploma	11 (6.7)
Diploma	59 (36.3)
Associate's degree	3 (8)
Bachelor's degree	55 (33.7)
Master's degree	22 (13.50)
PhD degree	3 (1.8)
Fathers' employment	
Government employee	67 (41.30)
Freelance job	95 (58)
Unemployed	1 (0.70)
Mothers' employment	
Government employee	30 (18.60)
Housewife	120 (73.30)
Freelance job	13 (8.10)

### 3.1 Parents' Knowledge, Attitudes, and Performance for Their Children's Oral Health

Table 2 shows a distribution of the attainable knowledge, attitude, and behavior scores for the variables. At the onset, 126 (77.30%) of the parents had a moderate level of knowledge (mean score: 9.71 ± 2.03). More than half of the parents were aware that brushing and dentifrices prevent tooth caries [132 (81%)] and knew when the first permanent teeth [112 (68.71%)] erupt. However, less than half of the participants agreed with the importance of the dentist's role in preventive dentistry [64 (29.3%)], use of dental floss [47 (28.8%)], and definition of dental plaque [25 (15.3%)]. The mean initial attitude score was 4.59 ± 1.02, of which 145 (89.00%) had a moderate attitude level. Only 6 (3.7%) had an appropriate attitude about the importance of preserving primary teeth. In total, 152 (93.20%) believed they were not responsible for preventing dental caries in their children. The mean of initial performance was 17.20 ± 4.05. From these, 131 (80.40%) indicated moderate performance. However, only 25 (15.30%) of the parents routinely took their children to a dentist (every 6–12 months). A total of 82 (50.30%) children brushed their teeth once a day and 32 (19.70%) brushed their teeth twice or more each day. Only 45 (27.60%) of the participants used dental floss. In addition, 136 (83.40%) consumed snacks between meals and 118 (72.40%) used cariogenic snacks once or more a day during this time.

**Table 2:** Parents' knowledge, attitudes, and practices toward oral health and the impact of COVID‐19 on these variables.

Variable	Attainable score		
	Poor (< 1/3), *N* (%)	Moderate (1/3–2/3), *N* (%)	Good (> 2/3), *N* (%)
Parents' knowledge about oral health	10 (6.10)	126 (77.30)	27 (16.6)
Parents' attitude about oral health	13 (8)	145 (89)	5 (3)
Parents' practice of oral health	28 (17.2)	131 (80.4)	4 (2.4)
Parents' knowledge of the probability of children being infected with the COVID‐19 virus in dental offices	14 (8.6)	31 (19)	118 (72.4)
Parents' attitude in terms of concern about COVID‐19	16 (9.8)	86 (52.8)	61 (37.4)
Parents' practice of children's oral health during the COVID‐19 pandemic	88 (54)	52 (31.9)	23 (14.1)

Neither knowledge nor attitude before the intervention had a significant association with demographic characteristics. Academic education for fathers (*p* = 0.002) and mothers (*p* = 0.002) and mother's employment (*p* < 0.001) were positively associated with initial performance score. Parents with one child had higher performance scores compared to those with two or more children (*p* = 0.040) (Table 3).

**Table 3:** Parents' knowledge, attitudes, and practices about oral health in children.

Variable	*N*	Mean ± SD					
		Knowledge	Attitude	Practice	Knowledge about COVID‐19	Attitude about COVID‐19	Practice during COVID‐19
Children's gender							
Male	75	9.60 ± 1.86	4.63 ± 1.15	17.68 ± 4.15	3.68 ± 1.18	1.58 ± 1.00	1.47 ± 0.99
Female	88	9.87 ± 2.21	4.53 ± 0.86	16.64 ± 3.87	3.56 ± 1.21	1.63 ± 1.18	1.60 ± 0.98
*p*		0.389	0.523	0.102	0.517	0.397	0.375
Number of children							
1	52	9.84 ± 2.00	4.61 ± 0.79	18.40 ± 3.27	3.40 ± 1.47	1.63 ± 1.15	1.62 ± 1.03
2	79	9.49 ± 1.97	4.60 ± 1.12	16.64 ± 4.52	3.74 ± 1.10	1.59 ± 0.99	1.52 ± 0.92
≥ 3	32	10.19 ± 2.08	4.51 ± 1.121	16.80 ± 3.50	3.70 ± 0.82	1.40 ± 1.22	1.36 ± 1.06
*p*		0.236	0.899	0.040	0.253	0.667	0.519
Fathers' education level							
Less than diploma	23	10.45 ± 2.58	4.50 ± 0.67	14.72 ± 4.50	3.40 ± 1.33	1.62 ± 1.12	1.31 ± 0.67
Diploma and higher	140	9.61 ± 1.92	4.60 ± 1.07	17.57 ± 3.85	3.65 ± 1.16	1.65 ± 1.08	1.54 ± 1.00
*p*		0.069	0.650	0.002	0.379	0.928	0.212
Mothers' education level							
Less than diploma	11	9.91 ± 3.18	4.27 ± 0.64	13.63 ± 5.14	3.36 ± 1.50	3.00 ± 1.18	1.50 ± 0.75
Diploma and higher	152	9.70 ± 1.93	4.61 ± 1.04	17.46 ± 3.85	3.64 ± 1.17	2.73 ± 1.07	1.53 ± 0.99
*p*		< 0.747	0.290	0.002	0.452	0.428	0.933
Fathers' job							
Government employee	67	9.77 ± 1.79	4.65 ± 0.98	18.00 ± 3.67	3.65 ± 1.26	1.68 ± 1.18	1.50 ± 1.04
Freelance	95	9.74 ± 2.27	4.56 ± 1.06	16.91 ± 4.16	3.59 ± 1.14	1.55 ± 1.01	1.54 ± 0.89
*p*		0.945	0.592	0.134	0.772	0.876	0.830
Mothers' job							
Employee (government or freelance)	43	9.74 ± 1.64	4.72 ± 1.18	16.09 ± 3.81	3.81 ± 1.07	1.61 ± 1.08	1.56 ± 1.11
Housewife	120	9.72 ± 2.16	4.53 ± 0.95	18.72 ± 4.02	3.55 ± 1.23	1.62 ± 1.09	1.52 ± 0.94
*p*		0.941	0.306	< 0.001	0.219	0.843	0.848

*Note: p* < 0.05 indicates statistical significance.

Abbreviations: *N*, number of participants; *p*, *p* value.

### 3.2 Results of the Variables During the COVID‐19 Pandemic

More than half of the parents [119 (72.9%)] showed good knowledge of COVID‐19 transmission in the dental setting, 86 (52.80%) had a moderate attitude about this disease, and 88 (54%) of the participants exhibited poor performance with regard to children's oral and dental health during the COVID‐19 pandemic.

In total, 87 (53.40%) reported a reduction in regular dental visits, and 49 (30%) used homecare treatment to relieve dental pain. Mean knowledge of COVID‐19 transmission in the dental setting, concern about the disease, and performance during the COVID‐19 pandemic were 3.63 ± 1.19, 1.62 ± 0.72, and 1.55 ± 0.97, respectively. Many participants [119 (72.90%)] agreed about the probability of transmission of COVID‐19 through the dental office. Also, over half of the parents expressed fear [90 (55.20%)] of COVID‐19 and concern about it. Only 9 (5.50%) parents used online or telephone consultations with their dentists. No significant association was found between demographic characteristics and mean scores for these variables (Table 4).

**Table 4:** Parents' knowledge, attitudes, and practices toward oral health before and after intervention.

Variable	Attainable score (mean ± SD)		*p*	*d*
	Before intervention	After intervention		
Parents' knowledge toward oral health	9.71 ± 2.03	12.26 ± 2.20	< 0.001	0.80
Parents' attitudes toward oral health	4.59 ± 1.02	8.99 ± 1.18	< 0.001	2.55
Parents' practices toward oral health	17.20 ± 4.05	21.17 ± 4.74	< 0.001	0.62
Parents' knowledge of the probability of children being infected with the COVID‐19 virus in dental offices	3.36 ± 1.19	3.90 ± 1.19	0.030	0.17
Parents' attitudes with regard to concerns about COVID‐19	1.55 ± 0.97	2.08 ± 1.20	< 0.001	0.34
Parents' practices in maintaining children's oral health during the COVID‐19 pandemic	1.62 ± 0.72	2.01 ± 1.00	< 0.001	0.33

*Note: p* < 0.05 indicates statistical significance.

Abbreviations: *d*, Cohen's effect; *p*, *p* value.

### 3.3 Correlations Between the Knowledge, Attitudes, and Performance Scores

Pearson's correlation coefficient results showed no significant pairwise association between parents' knowledge, attitudes, and performance concerning oral health (all *p* > 0.05). Similarly, the three corresponding scores of COVID‐19 were not significantly associated with each other (all *p* > 0.05). However, there was a significant correlation with acceptable effect size (|*r*| ≥ 0.4) between knowledge and attitude of COVID‐19 in the dental setting (*r* = 0.490, *p* < 0.001). Although we observed a significant correlation between knowledge and both attitude and performance during the pandemic, the sizes of the correlation coefficients were not remarkable.

### 3.4 Before and After Educational Intervention

Educational intervention resulted in a significant increase in mean knowledge, attitude, and performance scores in oral health (all *p* < 0.001) (Table 4). Cohen's *d* effect size revealed that the increase was large for knowledge (*d* = 0.80) and attitude (*d* = 2.55), but moderate for performance toward oral health (*d* = 0.62).

There was a significant increase after the intervention in mean knowledge about COVID‐19 transmission in the dental setting, concern about the disease, and performance compared to before the intervention (all *p* < 0.05). However, the effect sizes indicated a small to negligible increase for the three variables (all *d* < 0.5).

The intervention led to a change in performance scores among housewives, which was greater than working mothers (*p* = 0.004). Between 136 and 144 (83.40%–88.40%) participants believed that interviews and repetition could influence their opinions about oral health care. The use of snacks decreased in 73 (44.80%) children. The number of dental examinations increased in 50 (30.70%) cases.

## 4 Discussion

Parents play an important role in shaping their children's oral health behaviors (Poutanen et al. 2006). This study assessed the level of parents' knowledge, attitudes, and performance with respect to their children's oral health, and the impact of COVID‐19 on these variables. We also evaluated the effectiveness of intervention education on children's oral health. A questionnaire was used to assess these variables (Basir, Khanehmasjedi, and Khanehmasjedi 2022; Ceyhan, Kirzioglu, and Yildirim 2022; Dumitrescu et al. 2022; Llena et al. 2015).

The findings confirmed a previous study that showed a moderate level of parental awareness about children's oral health (Basir, Khanehmasjedi, and Khanehmasjedi 2022). In contrast to other reports (Hamasha et al. 2019; Saied‐Moallemi et al. 2008), our results showed that few parents knew about dental plaque and its consequences on oral diseases. Like others, the present study showed most parents were aware that brushing prevented tooth caries and knew when the first permanent teeth would erupt (Basir, Khanehmasjedi, and Khanehmasjedi 2022; Calcagnile et al. 2019; Saied‐Moallemi et al. 2008); this contradicted the findings reported by Zhu et al. (2003).

Parents' attitudes toward children's oral health were moderate, and this was in line with a prior study (Basir, Khanehmasjedi, and Khanehmasjedi 2022). There was no correlation between parental attitudes and educational level or occupation.

The present research indicated that parents' performance toward their children's oral health was moderate; only a few percent performed well. Higher educated parents displayed superior performance, which supported the results from other studies (Basir, Khanehmasjedi, and Khanehmasjedi 2022; Goodarzi et al. 2019; Pranno et al. 2022). However, two studies reported no association between parents' education and children's oral hygiene (Chen et al. 2020; Dumitrescu et al. 2022). Our results, like Basir, Khanehmasjedi, and Khanehmasjedi (2022), showed that less than one‐third of children brushed twice each day. Other studies reported that more children brushed twice daily (Saied‐Moallemi et al. 2008; Zhu et al. 2003). In agreement with a previous study, working mothers had better performance than housewives (Soltani et al. 2020). This may be related to income, which would give employed mothers the opportunity for enhanced oral health care for their children. The difference between the results may be due to the differences in the age of the participants, parental level of education and occupation, cultural differences, sample sizes, and survey methods (Ceyhan, Kirzioglu, and Yildirim 2022; Dumitrescu et al. 2022; Hamasha et al. 2019; Saied‐Moallemi et al. 2008; Zhu et al. 2003).

In the current study, many participants agreed on the probability of transmission of COVID‐19 through the dental office staff or other patients, which was in line with a previous study (Łazarz‐Półkoszek et al. 2023) but contrasted those reported by Ceyhan, Kirzioglu, and Yildirim (2022). Similar to Surme et al. (2021), we did not find an association between the knowledge of being infected by coronavirus in the dental environment and demographic information. Like others, our results showed that most of the parents expressed fear of COVID‐19 and concern about its spread or increasing numbers of illnesses (Ali et al. 2023; Martínez‐Lorca et al. 2020).

We noted a decline in parents' oral health performance throughout the COVID‐19 pandemic. Parents failed to monitor their children's oral hygiene and consumption of a non‐cariogenic diet. Like Farsi and Farsi (2021), regular dental appointments decreased due to the widespread closure of dental offices, self‐treatment at home, and parents' concerns about infection. Our data showed that some of the parents used home remedies to postpone dental appointments in accordance with Sen Tunc et al. (2021). School closings kept most children at home and our results showed that the consumption of cariogenic snacks increased. There is a relationship between the consumption of a cariogenic diet and dental caries in children (Llena et al. 2015). Therefore, it is possible that the pandemic may have increased the probability of dental caries in children, tooth pain, and a decreased quality of life depending on oral health (Samuel et al. 2021; Yang et al. 2021); this should be addressed in future studies.

The use of teledentistry (phone calls and text messaging) is a promising strategy for maintaining communication with patients without exposure to infection, and this strategy enables parents to distinguish between urgent and nonurgent situations (Yang et al. 2021). The initial survey showed that few parents used online or telephone consultations with dentists. Our findings were lower than those reported by Rahman, Nathwani, and Kandiah (2020). This may be related to the lack of adequate infrastructure, public awareness, and the use of technology in each society (Ghai 2020).

In this study, the health educational intervention was conducted by face‐to‐face teaching with appropriate tools. The training delivered in the interview sessions was repeated over the phone or by video call. Our findings revealed that health educational intervention significantly improved the level of the study variables, which agreed with prior studies (Halawany et al. 2018; Makvandi et al. 2015; Vozza et al. 2017, 2019). In contrast, Alrashdi et al. (2021) did not report a significant change in the families following educational intervention.

Potential limitations of this study included the difficult access to the participant group during the pandemic. Although the present study was limited by the self‐reporting methodology, we used a comprehensive, user‐friendly questionnaire and face‐to‐face and teledentistry methods. Also, this research was the first study that assessed the influence of the COVID‐19 pandemic on the various aspects of parents in terms of children's oral health. Another highlight of this study was the availability of a dentist to answer parents' questions during the research. Notably, cessation of the COVID‐19 pandemic does not negate the value of the data collected during this unprecedented global health event. These data provide a critical baseline for understanding the shifts in parental behavior regarding children's oral health and highlight areas where health care systems may need to adapt to ensure better outcomes. The persistence of these changes post‐pandemic suggests that the impact of such a global event continues to influence health care decisions and outcomes long after its resolution. Future research is recommended with a larger patient population along with clinical examinations.

## 5 Conclusions

The awareness, attitudes, and performance of parents toward oral and dental health in children were graded as moderate. During the COVID‐19 pandemic, parents had good knowledge, a moderate attitude, and poor performance. These variables increased dramatically after the educational intervention program. We recommend in such crises that continuing education should be provided to parents along with preventive and therapeutic programs to address children's oral and dental health needs.

## Author Contributions

M.M., F.R., and A.R. conceptualized and designed the study, interpreted the data, drafted the manuscript, and approved the final manuscript as submitted. F.F. contributed to the conceptualization and design of the study, data collection, initial analyses, manuscript review, and approval of the final manuscript as submitted. M.V. was involved in the conceptualization and design of the study, data acquisition and analysis, critical revision of the manuscript for important intellectual content, and approval of the final manuscript as submitted. All authors approve the final manuscript as submitted and agree to be accountable for all aspects of this work.

## Ethics Statement

The research protocol was approved by the Human Ethics Review Committee of the School of Dentistry, Shiraz University of Medical Sciences (ID IR.SUMS.DENTAL.REC.1399.220).

## Conflicts of Interest

The authors declare no conflicts of interest.

## Supporting information

APPENDICES: Questionnaire file.

## Data Availability

The data that support the findings of this study are available from the corresponding author upon reasonable request.
