# Supplementary material for: Impact of the COVID‐19 Pandemic on Oral Health Behaviors Among Children in Iran
Source: Clin Exp Dent Res. 2024 Nov 11;10(6):e70010. doi: 10.1002/cre2.70010 (PMC11551591; doi:10.1002/cre2.70010)
Supplement: Supplementary file 1 — APPENDICES: Questionnaire file. [file CRE2-10-e70010-s001.docx]

**Additional file 1: Questionnaire of the impact of the COVID-19 pandemic on oral health behaviors among children.**

| Dear Parents, the following research project investigates the impact of the COVID-19 pandemic on oral health behaviors among children. We would appreciate it if you would help us by completing this questionnaire. The information will be used in a cross-sectional study and your data will be confidential. Completing this questionnaire would make you a participant in this study. Thank you for your sincere cooperation.  Location when completing the form: ....... Residence area: ...... Date of completing the form: 2021 /…/… |
| --- |

| **Demographic information of the respondent** |
| --- |
| Gender: Boy🞎 Girl🞎 Date of child's birth: ... /... / ...,  Date of father's birth ... / ... / ... Date of mother's birth: ... / ... /...  Number of children: ............... **What is your child’s birth order**............  Father's education: ................. Father's job: .................  Mother's education: ................. Mother's job: .......................... |

| **Parents' knowledge about children's oral health** | **Strongly agree** | **Agree** | **No comment** | **Disagree** | **Strongly disagree** |
| --- | --- | --- | --- | --- | --- |
| 1. I should care for my child's teeth in addition to the rest of his/her body |  |  |  |  |  |
| 2. Sweets cause cavities and destroy teeth |  |  |  |  |  |
| 3. Caries would make it hard to chew and make the smile look unpleasant |  |  |  |  |  |
| 4. Tooth brushing prevents dental caries |  |  |  |  |  |
| 5. Fluoride dentifrices prevent dental caries in children |  |  |  |  |  |
| 6. Mouthwash is an effective method to reduce oral microorganisms |  |  |  |  |  |
| 7. There's no need to floss every day |  |  |  |  |  |
| 8. Periodic and regular dental appointments are required |  |  |  |  |  |
| 9. Parents can evaluate their child's mouth and determine the next dental appointment |  |  |  |  |  |
| 10. The dentist's role is limited to tooth treatment and not prevention |  |  |  |  |  |
| 11. Some fruits cause dental caries |  |  |  |  |  |
| 12. For how many minutes do the teeth need to be brushed?  A) Less than one minute B) One minute C) Two minutes D) More than two minutes | | | | | |
| 13. Which condition causes gum bleeding?  A) Gingiva recession B) Inflamed gingiva C) Healthy gingiva D) I do not know | | | | | |
| 14. What does plaque mean?  A. Soft deposits on the teeth B. Heavy deposits on the teeth  C. Stains on the teeth D. I do not know | | | | | |
| 15. Influence of dental plaque:  A. Induces dental caries B. Induces periodontal diseases  C. Induces dental caries and periodontal diseases D. I do not know | | | | | |
| 16. 16. The first primary teeth erupt at the age of ………..and permanent teeth erupt at the age of ...................... | | | | | |

| **Parent’s attitudes about children's oral health** | **Strongly agree** | **Agree** | **No comment** | **Disagree** | **Strongly disagree** |
| --- | --- | --- | --- | --- | --- |
| 17. There is not much I can do to prevent my child's teeth from getting caries |  |  |  |  |  |
| 18. Deciduous teeth are temporary teeth and do not need to be preserved |  |  |  |  |  |
| 19. Children's dental problems can be serious |  |  |  |  |  |
| 20. If one of my relatives suffer from tooth problems, I would recommend they visit a dentist |  |  |  |  |  |
| 21. I am frightened of dentists |  |  |  |  |  |
| 22. I am only concerned about my child’s permanent teeth and take care of them |  |  |  |  |  |
| 23. Parent’s tooth brushing is a good model for teaching oral health to their children |  |  |  |  |  |
| 24. Parents are not responsible for their children's oral health |  |  |  |  |  |
| 25. Parents are only responsible for monitoring the child's dental pain and treatment |  |  |  |  |  |
| 26. Parents are responsible for controlling their children's consumption of sugary food |  |  |  |  |  |

| **Parent’s performance about children's oral health** | **Always** | **Often** | **Sometimes** | **Never** |
| --- | --- | --- | --- | --- |
| 27. I regularly check my child's teeth |  |  |  |  |
| 28. If I observe cavities in my child's teeth, I won't take them to the dentist until they have pain |  |  |  |  |
| 29. My child uses mouthwash every week |  |  |  |  |
| 30. My child uses dental floss every day |  |  |  |  |
| 31. I use children’s toothpaste to brush my child's teeth |  |  |  |  |
| 32. Using of toothpicks is not suitable for children |  |  |  |  |
| 33. I know the causes of tooth decay, but I cannot prevent it |  |  |  |  |
| 34. How often does your child visit a dentist?  A) Regular (every 6 to12 months) B) Occasionally  C) Whenever they have a toothache D) Has not been examined yet | | | | |
| 35. As a parent, what do you do when your child is brushing his/her teeth? This question may have several answers.  A) I supervise my child's brushing method B) We recommend brushing but we do not supervise  C) We do not pay attention at all D) My child allows me to brush his/her teeth | | | | |
| 36. What time does your child brush his/her teeth? This question may have several answers.  A) In the morning B) Before sleeping at night C) After lunch D) Other times (please name) | | | | |
| 37. What tools does your child use to clean his/her teeth? This question may have several answers.  A) Toothbrush and toothpaste B) Dental floss  C) Mouthwash D) Other items (please name) | | | | |
| 38. How often does your child consume snacks? This question may have several answers  A) More than once a day B) Once a day C) Once a week D) Never | | | | |
| 39. What is the most frequent drink (other than water) your child consumes daily or three to four times a week?  A) Soda B) Milk C) Ready-made packaged juices D) Cola or energy drink | | | | |
| 40. When does your child eat more snacks during the day?  A) With lunch or dinner B) Between meals C) Before bed D) Every time | | | | |
| 41. How often does your child brush his/her teeth?  A) Once a day B) Twice a day C) More than twice a day D) Every few days | | | | |
| 42. How often do you give your child cariogenic snacks (fermentable carbohydrates):  A) I give snacks to my child whenever he/she wants B) I give snacks according to his/her diet plan  C) I give snacks whenever he/she cries D) I give snacks to my child whenever he/she is hungry | | | | |
| 43. What was your child’s feeling during the first meeting with the dentist?  A) He was scared and did not want to be treated B) He was scared  C) He was a little afraid D) Not afraid | | | | |
| 44. What was the last dental treatment for your child prior to the onset of COVID-19?  A. Examination or radiography B. Fluoride therapy or sealant therapy  C. Pulp therapy D. Other treatments (tooth extraction, dental abscess or trauma) | | | | |
| 45. What is the most consumed snack?  A) Cookies and cakes B) Chocolate C) Chips and pastel candy  D) Ice cream E) Nuts F) Other items (Please name) | | | | |
| 46. If your child consumes fruit as a snack, which fruits does he/she use the most (2 items)? | | | | |
| 47. What was the reason for your child's last dental visit prior to the onset of COVID-19?  A. Toothache B. Tooth restoration C. Scaling  D. Periodic monitoring of oral health E. I have never visited a dentist | | | | |
| 48. What is the reason for your child's fear of the dentist? This question may have several answers.  A) Sound of the dental instruments B) Injection for anesthesia  C) Pain during treatment D) Distance to the dentist  Q) Having an appointment with the dentist the next day I) Sitting in the waiting room | | | | |

| **Parents' knowledge of Covid-19 disease (Coronavirus)** | **Strongly agree** | **Agree** | **No comment** | **Disagree** | **Strongly disagree** |
| --- | --- | --- | --- | --- | --- |
| 49. Children have a lower possibility of having COVID-19 |  |  |  |  |  |
| 50. There is a chance of transmitting the corona virus infection to a child in any place |  |  |  |  |  |
| 51. There is a chance of the child being exposed to the virus through the medical environment |  |  |  |  |  |
| 52. There is a chance of the child being exposed to the virus through other patients |  |  |  |  |  |
| 53. There is a chance of the child being exposed to the virus through the dental staff |  |  |  |  |  |

| **Parents' attitudes toward dentistry during**  **COVID-19 pandemic (Coronavirus)** | **Strongly agree** | **Agree** | **No comment** | **Disagree** | **Strongly disagree** |
| --- | --- | --- | --- | --- | --- |
| 54. I am always afraid of COVID-19 |  |  |  |  |  |
| 55. I get anxious when I see or hear the news about COVID-19 |  |  |  |  |  |
| 56. My heart rate rises when I think about having COVID-19 |  |  |  |  |  |

| **Parents' performance for dentistry during the**  **COVID-19 pandemic (Coronavirus)** |
| --- |
| 57. How did children’s compliance with oral hygiene (brushing teeth, using dental floss) change compared to before COVID-19?  A) It has increased B) It has decreased C) It did not change |
| 58. As a parent, how has your monitoring of your child's oral health (tooth brushing) changed compared to before COVID-19?  A) It has increased B) It has decreased C) It did not change |
| 59. Since the beginning of the COVID-19 pandemic, how has the number of snacks consumed by children during the quarantine at home changed?  A) It has increased B) It has decreased C) It did not change |
| 60. How has the rate of periodical referrals for children's dental treatments changed since the start of the pandemic?  A) It has increased B) It has decreased C) It did not change |
| 61. If your child's visits to the dentist have decreased since the beginning of the pandemic, what is the reason? This question may have several answers.  A) This period has led to better oral health behaviors (brushing, etc.) in children  B) This period has led to better supervision and encouragement of parents on oral health behaviors (brushing, etc.) in children  C) This period has limited the consumption of sweet and harmful snacks for the health of children's teeth.  D) I used online or telephone consultation with a dentist  E) I used home treatment to delay going to the dentist |
| 62. What was the main reason for taking your child to the dentist for treatment during the COVID-19 outbreak?  A) Normal treatments B) Emergency treatment only (toothache, infection, abscess, etc.)  C) Despite the need for emergency treatment, I did not go to the dentist due to concerns about COVID-19 exposure  D) I wanted to go but the medical centers were closed |
